# NEXThaler*^®^* in Focus: Evaluating Inhalers’ Expectations, Experiences, and Preferences Across Patients, General Practitioners, and Specialists

**DOI:** 10.3390/jcm14228070

**Published:** 2025-11-14

**Authors:** Piotr Damiański, Maciej Wojakiewicz, Tomasz Dębowski, Wojciech Jerzy Piotrowski, Adam Jerzy Białas

**Affiliations:** 1 Clinical Department of Internal Medicine, Asthma and Allergy, Medical University of Lodz, 90-419 Lodz, Poland; 2 Department of Pneumology, Medical University of Lodz, 90-419 Lodz, Poland; wojciech.piotrowski@umed.lodz.pl (W.J.P.); adam.bialas@umed.lodz.pl (A.J.B.); 3 Medical Department, Chiesi Poland Sp. z o.o., 02-305 Warsaw, Poland; m.wojakiewicz@chiesi.com (M.W.); t.debowski@chiesi.com (T.D.)

**Keywords:** asthma and COPD management, inhaler preference, NEXThaler^®^, patient–physician perspectives

## Abstract

**Introduction:** Choosing the optimal inhaler is crucial for effective management of asthma and COPD. Preferences and experiences may differ between patients and healthcare providers. The NEXThaler^®^, a dry powder inhaler (DPI), was developed to simplify inhalation and improve patient satisfaction. **Aim:** This survey examined factors influencing inhaler selection and compared perceptions of NEXThaler^®^ with currently used or prescribed inhalers (CUI/CPI) among patients, general practitioners (GPs), and specialists. **Methods:** A cross-sectional survey was conducted in Poland among 96 patients with asthma or COPD and 151 physicians (70 GPs, 81 specialists). Participants assessed inhaler attributes using MaxDiff scaling and rated their CUI/CPI and NEXThaler^®^ on a Likert scale to evaluate usability and satisfaction. **Results:** Both patients and physicians prioritized ease of inhalation and confidence in administering the correct dose. Patients rated these highest (means 11.09; 95% CI 10.16–12.02 and 9.83; 95% CI 8.87–10.79), similar to physicians (12.35; 95% CI 11.76–12.94 and 15.41; 95% CI 14.78–16.04). Device size and clarity of instructions had a minimal impact on inhaler choice (patients: 3.44; 95% CI, 2.93–3.95 and 3.64; 95% CI, 2.82–4.46; physicians: 0.91; 95% CI, 0.58–1.24 and 1.60; 95% CI, 1.28–1.92). For CPI, specialists rated devices higher than GPs in terms of quality, feedback systems, clarity of manuals, and confidence that patients use the inhaler correctly (*p* < 0.05). For NEXThaler^®^, both groups gave comparable, high scores (median 5 [4–5]; *p* > 0.05). Patients rated NEXThaler^®^ higher than their CUI for innovation, ease of use, dose counter, and feedback features (*p* < 0.001). Overall, 79% of healthcare providers and 71% of patients preferred NEXThaler^®^. **Conclusions:** Both patients and healthcare providers evaluated NEXThaler^®^ positively, particularly regarding usability and dose control. However, the results also highlight ongoing gaps in inhaler-related knowledge and confidence, especially in primary care, emphasizing the need for continued education and collaborative training to improve the effectiveness of inhalation therapy.

## 1. Introduction

In contemporary healthcare, patient-centered care has become essential in enhancing treatment outcomes for individuals with respiratory diseases. This approach emphasizes shared decision-making, taking into account patients’ preferences, concerns, and expectations. Bridging the gap between physician and patient perspectives is crucial, as it leads to effective, simplified, and individually tailored treatment regimens [1,2]. Accurately identifying and integrating both patient and physician expectations in daily clinical practice—whether in inhaler selection or in the choice of advanced therapies such as biologics—is vital. A clear understanding of these preferences can reduce inhalation errors, enhance adherence to medical guidelines, optimize therapeutic outcomes, and increase patient satisfaction [2,3,4,5].

The current market offers hundreds of inhaler models varying in type, brand, and design, providing extensive personalized therapeutic options. However, this diversity also introduces significant challenges, as the complexity of choosing an appropriate device can lead to confusion among healthcare professionals (HCPs) and patients alike [6]. Consequently, incorrect inhalation technique is widespread; up to 90% of patients make some technical errors regardless of inhaler type. This is especially pertinent given evidence from a meta-analysis of 59 randomized studies demonstrating minimal differences in therapeutic efficacy among various inhalation devices (pressurized metered-dose inhalers (pMDIs), dry powder inhalers (DPIs), and nebulizers) when used correctly [7,8]. Educational interventions significantly improve inhalation techniques across all inhaler types, although the effectiveness of training, particularly for pMDIs, depends to some extent on selecting an instructional method appropriate to the specific inhaler type [9].

Importantly, perceptions of ideal inhaler characteristics often differ between patients and healthcare professionals, and these differences can vary among medical specialties. Physicians, influenced by clinical efficacy and technical considerations, might prioritize different device features than patients, who typically emphasize ease of use, convenience, and practical everyday applicability. Therefore, inhalers incorporating intuitive functionality, clear instructional materials, and patient-centered design features have the potential to enhance both clinical outcomes and user satisfaction significantly [10].

The NEXThaler^®^, a DPI containing extrafine particles of beclometasone dipropionate/formoterol (BDP/FF), has been developed specifically to simplify the inhalation process and address common problems encountered with existing inhalers. Its practical features include a breath-activated mechanism (BAM), releasing medication only at inspiratory flows equal to or above 35 L/min, thus minimizing large particle emissions and enhancing deep pulmonary deposition of extrafine particles. The NEXThaler^®^ also employs a triple feedback system—consisting of a clear dose counter, lactose taste confirmation, and an audible “click” upon activation—to reinforce proper inhalation technique. The device has been shown to deliver consistent doses across a broad inspiratory flow range (35–90 L/min), which may support its use in patients with different respiratory capacities and levels of asthma control [11,12,13]. However, despite these technical advantages, the clinical relevance and comparative benefit of this device over other DPIs remain under debate. Some studies showed that advanced feedback systems and the use of extrafine particles may offer certain advantages in terms of device usability and drug delivery efficiency. Nevertheless, evidence suggests that aspects such as education on correct inhalation technique, patient preference, and adherence may play a more decisive role in determining clinical outcomes than the specific design of the inhaler itself [6,14,15].

Considering these challenges and future perspectives in inhalation therapy, the article provides a comprehensive analysis of key factors influencing inhaler selection from both patient and physician perspectives, with particular focus on evaluating the NEXThaler^®^ dry powder inhaler compared to other commonly used or prescribed inhalation devices (CUI/CPI). To achieve this, a cross-sectional survey was conducted in Poland among patients with asthma or chronic obstructive pulmonary disease (COPD) and physicians (general practitioners (GPs), pulmonologists, and allergologists) to assess and compare their perceptions, experiences, and preferences regarding inhaler use.

## 2. Methodology

### 2.1. Survey Design and Data Collection

This cross-sectional survey was conducted between 19 December 2021 and 11 February 2022 in Poland, among patients diagnosed with asthma or COPD, as well as physicians, including (GPs), pulmonologists, and allergologists. The current analysis was based on these archival survey data collected by Ipsos on behalf of Chiesi. At the time of data collection, the NEXThaler^®^ device was not yet commercially available in Poland. Consequently, none of the participants had prior experience with this specific inhaler.

Participants were recruited via professional interviewers and the Ipsos Medical Panel. Before completing the questionnaire, all respondents received standardized instructions from a member of the research team on how to complete it. Participants were asked to answer independently and honestly and were subsequently provided with a secure link to an online questionnaire via email.

The survey employed a structured, quantitative design using the Computer-Assisted Web Interviewing (CAWI) methodology. Only fully completed questionnaires were included in the analysis, and no cases of dropout or partial responses were reported. Initially, participants completed a set of questions evaluating their expectations and preferences regarding the ideal characteristics of an inhaler, including aspects such as usability, ease of training, device functionality, and confidence in correct use. Following this, respondents were asked to evaluate either their currently used inhaler (CUI) (patients) or inhalers they typically prescribe (physicians) (CPI), based on the presented inhaler attributes. Lastly, all participants were presented with the placebo NEXThaler^®^ DPI, which included a visual inspection, an inhalation trial, accompanying instructional materials, and a video demonstration of device use. To minimize potential presentation bias, all participants were shown the same standardized instructional video. The material provided a neutral and factual overview of NEXThaler^®^ features, without promotional language or comparative statements. They were then asked to assess the NEXThaler^®^ in direct comparison to the previously evaluated inhaler.

### 2.2. Inclusion Criteria

Patients:

Adults aged 18 years or older diagnosed with obstructive respiratory disease (asthma or COPD) were included.

Patients must have been receiving inhalation therapy for at least three months before participation.

Participants provided voluntary informed consent after receiving comprehensive information about the survey objectives, data processing policies, and privacy guidelines.

Physicians:

Licensed medical practitioners actively involved in treating patients with obstructive respiratory diseases (asthma, COPD, overlap phenotype) were included.

Physicians voluntarily agreed to participate after receiving detailed information about the purpose of the survey, data protection protocols, and privacy policy.

### 2.3. Exclusion Criteria

Lack of consent: Individuals who declined to participate or withdrew consent before completing the survey;

Lack of inhalation therapy: Patients who had not used inhalation therapy continuously for at least three months before the survey;

Other medical conditions: Patients suffering from severe comorbidities or psychiatric disorders may potentially influence the reliability of their responses or assessments.

### 2.4. Questionnaires

The survey sample included physicians (GPs and specialists) and patients with a confirmed diagnosis of asthma or COPD. Physicians provided information on their clinical practice, including average patient volume, their role in initiating or continuing inhalation therapy, years of professional experience, healthcare setting, and the types of inhalers most frequently used in clinical management. Patients reported demographic characteristics (age, sex) and details concerning their current use of inhalation therapy.

To investigate the relative importance of specific inhaler attributes, a MaxDiff (Maximum Difference Scaling) task was conducted separately for physicians and patients. The list of attributes was developed during the study design phase and refined through a qualitative pre-test conducted by the Ipsos Healthcare research team, which provided content and face validation to ensure clarity, clinical relevance, and comprehensiveness from both perspectives. During the task, respondents were shown sets of five attributes at a time (11 sets for physicians and 9 for patients) and asked to indicate the most and least important feature in each set. Individual-level utility scores, summing to 100 per respondent, were estimated using a hierarchical Bayesian model and then aggregated to compute mean preference scores. Questions assessing inhaler satisfaction and specific device features were developed by the Ipsos Healthcare team, based on internal expertise and insights from the qualitative phase preceding the quantitative survey.

In a subsequent section of the questionnaire, participants were asked to evaluate inhalers in use. Patients assessed their CUI, while physicians evaluated the CPI. Both groups were then presented with the NEXThaler^®^ and asked to rate it using the same set of features. Each characteristic was rated on a five-point Likert scale (5 = very, 4 = fairly, 3 = somewhat, 2 = not very, 1 = hardly at all), enabling structured comparisons between real-world inhalers and the NEXThaler^®^ in terms of usability, performance, and perceived value.

In addition, participants were asked to compare the NEXThaler^®^ directly with their CUI/CPI across a subset of predefined features (e.g., clarity of instructions, ease of use, perceived quality, overall preference). For each feature, respondents indicated which device they considered superior. The proportion of participants preferring the NEXThaler^®^ was calculated separately for patients and physicians to identify perceived advantages of the device across user groups.

### 2.5. Ethical Statement

This study was conducted in accordance with the principles of the Declaration of Helsinki. As this work involved a secondary analysis of anonymized survey data collected earlier by the independent research agency Ipsos, ethical approval was obtained after data collection from the Bioethics Committee of the Medical University of Lodz (approval no. RNN/07/25/KE, issued on 14 January 2025). All data were processed in compliance with applicable data protection regulations. Informed consent was obtained from all participants during the original data collection, and no personally identifiable information was accessed or used in the present analysis.

#### Data Handling and Anonymization

The secondary analysis was performed on anonymized data provided by Ipsos. All personal identifiers were removed before data transfer, and each respondent was assigned a numeric code to ensure confidentiality. The research team had access only to aggregated, de-identified data. All procedures complied with GDPR and the ethical standards approved for secondary data use.

### 2.6. Statistical Methods

Statistical analysis of the data was conducted using R software v.4.4.2 for macOS. Data normality was verified with the Shapiro–Wilk test. Continuous variables are reported as means with standard deviations (SDs) or medians with interquartile ranges (IQRs) based on the data distribution.

The MaxDiff data were analyzed using a hierarchical Bayesian estimation model, which allowed for the calculation of individual-level utility scores and aggregating mean preference scores for each inhaler attribute. The Wilcoxon rank-sum test with continuity correction was applied for two-group comparisons. Categorical data were analyzed using the chi-square test or Fisher’s exact test, as appropriate.

Statistical significance was defined as *p* < 0.05. Missing data were not imputed. No correction for multiple comparisons was applied, as all comparisons were pre-specified before the analyses, and all results, significant or not, are reported for transparency.

## 3. Results

### 3.1. Participant Characteristics and Inhaler Use Patterns (Table 1 and Table 2)

A total of 151 physicians participated in the survey, including 70 general practitioners (GPs) and 81 specialists (pulmonologists and allergologists). The majority were female (66%). A total of 19% of physicians were aged ≤45 years, 47% were between 46 and 55 years, and 34% were older than 55 years. Notable differences were observed between GPs and specialists. GPs reported caring for a significantly smaller number of patients with asthma or COPD per month than specialists (mean 75.8 vs. 150.7, *p* < 0.05). They also initiated inhalation therapy less frequently (17.66% vs. 26.99%, *p* < 0.05), but were more likely to continue ongoing treatment (82.4% vs. 73.0%, *p* < 0.05). Dry powder inhalers were the most commonly prescribed inhaler type in both groups (66% of GPs and 64% of specialists), followed by pressurized metered-dose inhalers. Soft mist inhalers were used exclusively by specialists (6%), with no reported use among GPs (*p* < 0.05).

**Table 1:** Characteristics and comparison of general practitioners (GPs) and specialists in the management of asthma and COPD: patient care, experience, and inhaler preferences.

		GPs (70)	Specialist (81)	*p*-Value
1. How many patients diagnosed with asthma/COPD do you typically care for a month?	M (SD)	75.8 (107.4)	150.7 (127.5)	<0.05
2. (%) of patients with asthma or COPD for whom I initiate therapy with an inhaled medication.	M (SD)	17.7 (13.25)	27 (16.12)	<0.05
3. (%) of patients with asthma and/or COPD for whom I continue ongoing therapy with an inhaled medication.	M (SD)	82.4 (13.25)	73 (16.12)	<0.05
4. How long have you practiced in this specialty?				
<15 years	%	31	38	0.37
16–20 years	%	40	22	<0.05
>20 years	%	29	40	0.17
5. Place of work				
Only inpatient care/hospital ward	%	0	0	N/A
Only outpatient care/clinic	%	71	36	<0.001
Both outpatient and inpatient care	%	29	64	<0.001
6. Which types of inhalers do you use most often in treating asthma or COPD?				
Dry powder inhalers	%	66	64	0.80
Pressurized inhalers	%	34	30	0.59
Soft mist inhalers	%	0	6	<0.05

Data are presented as the mean: M (SD), or (%). Abbreviations: COPD, chronic obstructive pulmonary disease, GP, general practitioners.

**Table 2:** Patients’ demographic data and inhalation therapy.

	Total	Asthma (79)	COPD (17)	*p* Value
**Age M**	48	46	54	<0.05
**Female (%)**	73	71	82	0.56
**Which types of inhalers do you use most often to treat asthma or COPD?**				
**Dry powder inhalers (%)**	50	52	47	0.59
**Pressurized inhalers (%)**	45	47	35	0.28
**Soft mist inhalers (%)**	5	1	18	<0.05

Data are presented as the mean: M, or (%). Abbreviations: COPD, chronic obstructive pulmonary disease.

A total of 96 patients who had used inhalation therapy in the three months prior to the survey were also included. Among them, 79 had asthma and 17 had COPD. The patient sample was predominantly female (73%). Regarding age, 4% were aged ≤45 years, 23% were aged 46–55 years, and 33% were older than 55 years. Patients with COPD were significantly older than those with asthma (mean age 54 vs. 46 years, *p* < 0.05). Across both patient groups, dry powder inhalers were the most commonly reported (50%), followed by pressurized inhalers (45%). Soft mist inhalers were significantly more prevalent among COPD patients than those with asthma (18% vs. 1%, *p* < 0.05).

### 3.2. Evaluation of the Key Characteristics of Inhalers from the Perspective of Physicians and Patients—MAXDIFF Analysis (Figure 1 and Figure 2)

**Figure 1:**
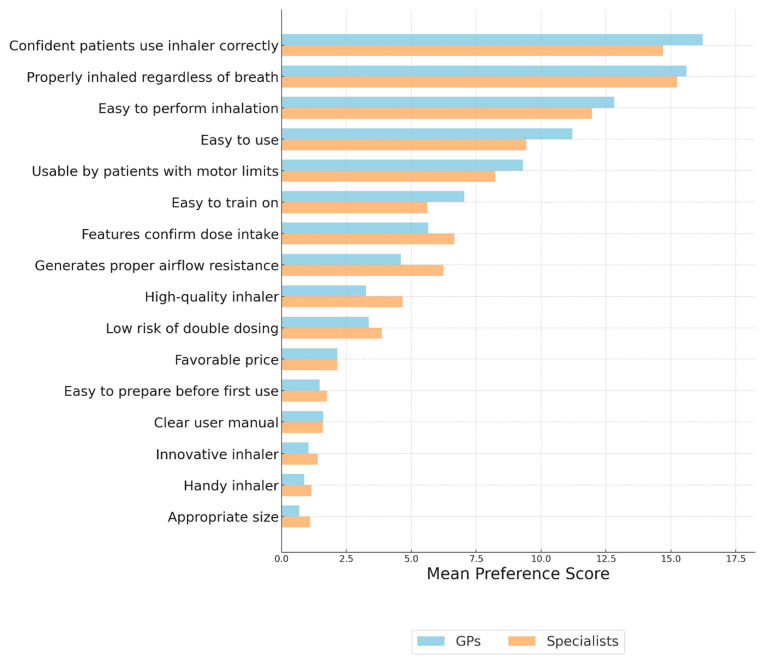
Evaluation of the key characteristics of inhalers from the perspective of general practitioners and specialists. Importance of Inhaler Features—MAXDIFF Analysis: Question: Among the following features, please select the one that is the most important and the one that is the least important when using an inhaler.

As shown in Figure 1, both GPs and specialists prioritized confidence that patients use the inhaler correctly and the ability to ensure proper medication inhalation regardless of the patient’s inspiratory capacity. GPs placed relatively higher importance on ease of use and ease of performing inhalation, whereas specialists assigned greater weight to technical features such as airflow resistance and confirmation of dose intake. Both groups considered some characteristics, such as favorable price, manual clarity, and device size, as less important.

**Figure 2:**
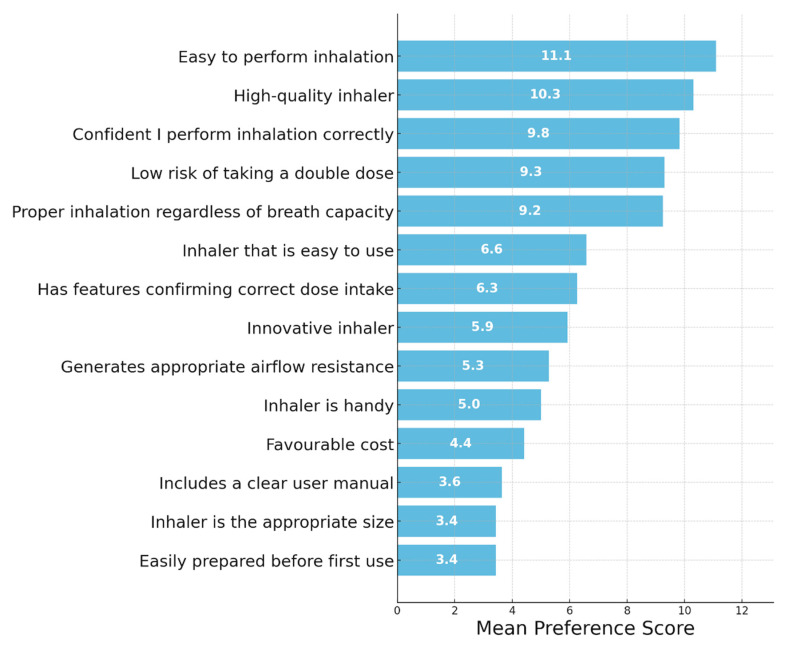
Evaluation of the key characteristics of inhalers from the perspective of patients. Importance of Inhaler Features—MAXDIFF Analysis: Question: Among the following features, please select the one that is the most important and the one that is the least important when using an inhaler.

Figure 2 presents results from the patient sample. Patients’ most highly valued features related to functionality, such as ease of performing inhalation, high quality of the device, and confidence in correct use. They also emphasized the importance of avoiding double dosing and ensuring proper inhalation regardless of inspiratory ability. Aesthetic and handling-related features were assigned lower preference scores, including size, user manual clarity, and ease of preparation before first use.

### 3.3. Comparison of Inhaler Characteristics: Currently Prescribed Inhaler vs. NEXThaler^®^ Across GP and Specialists (Table 3, Figure 3a,b)

**Figure 3:**
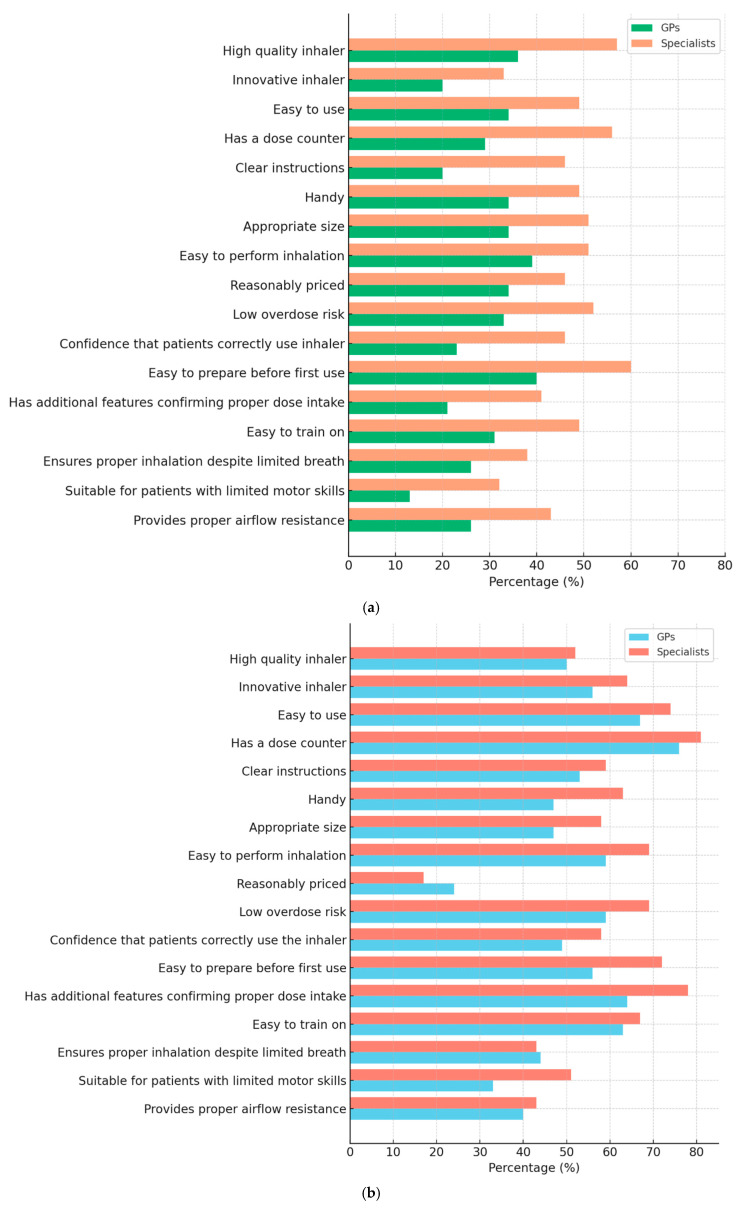
(**a**,**b**). Percentage of general practitioners (GPs) and specialists who rated each characteristic of their currently prescribed inhaler (**a**) and NEXThaler^®^ (**b**) as 5 (highest score) on a 5-point Likert scale. The figure compares perceptions of inhaler quality, usability, and functionality between the two physician groups.

The evaluation of CPI revealed that specialists rated the inhalers more favorably across most attributes compared to GPs, as shown in Figure 3. Features such as “High-quality inhaler” (57% vs. 36%), “Has a dose counter” (56% vs. 29%), and “Confidence that patients perform inhalation correctly” (46% vs. 23%) showed particularly notable differences (*p* < 0.05). Overall, satisfaction levels for CPI remained moderate in both groups.

In contrast, the NEXThaler^®^ inhaler received significantly higher ratings from GPs and specialists (Figure 3b). Particularly high ratings were seen for “Dose counter” (GPs 76%, specialists 81%), “Easy to use” (GPs 67%, specialists 74%), and “Has additional features confirming correct medication intake” (GPs 64%, specialists 78%).

Statistical analyses confirmed these findings. For currently prescribed inhalers, there were significant differences (*p* < 0.05) between GPs and specialists in most inhaler characteristics (e.g., high quality, innovation, ease of use, clear manual, dose counter, handy size, ease of inhalation), as shown in the Likert scale (1–5) (Table 3). Specialists consistently rated inhalers higher than GPs. In contrast, for NEXThaler^®^, the ratings between GPs and specialists were comparable, with no statistically significant differences for the majority of attributes (*p* > 0.05), except for being “handy” (*p* = 0.030) and “easy to prepare before first use” (*p* = 0.031), where specialists rated NEXThaler^®^ slightly higher.

Moreover, when comparing NEXThaler^®^ to CPI within the same group (GPs or specialists), NEXThaler^®^ achieved significantly higher median ratings for most features (indicated by * or ** in the medians). This included major improvements in perceived innovation, ease of use, confirmation of correct medication intake, and suitability for patients with motor limitations (*p* < 0.001).

**Table 3:** Currently prescribed inhaler vs. NEXThaler^®^ across GP and specialists.

Inhaler Characteristics	Currently Prescribed Inhaler		*p* Values	NEXThaler^®^		*p* Values
	GPs (70)	Specialists (81)		GPs (70)	Specialists (81)	
1. High-quality inhaler	4 [3–5]	5 [4–5]	<0.05	4.5 [4–5]	5 [4–5]	0.74
2. Innovative inhaler	3 [3–4]	4 [4–5]	<0.05	5 [4–5] **	5 [4–5] **	0.36
3. Inhaler that is easy to use	4 [4–5]	4 [4–5]	0.07	5 [4–5] **	5 [4–5] **	0.25
4. Dose counter	4 [3–5]	4 [4–5]	<0.001	5 [5–5] **	5 [5–5] **	0.38
5. Includes a clear user manual	4 [3–4]	4 [4–5]	<0.001	5 [4–5] **	5 [4–5] **	0.24
6. The inhaler is handy	4 [3–4]	4 [4–5]	<0.05	4 [4–5]	5 [4–5]	0.03
7. The inhaler is the appropriate size	4 [3.25–5]	5 [4–5]	<0.05	4 [4–5]	5 [4–5]	0.14
8. Easy to perform inhalation	4 [3.25–5]	5 [4–5]	<0.05	5 [4–5] *	5 [4–5] *	0.15
9. Low risk of taking a double dose of the medication	4 [3–5]	5 [4–5]	<0.05	5 [4–5] **	5 [4–5] *	0.22
10. I am confident that patients perform inhalation correctly	4 [3–4]	4 [4–5]	<0.001	4 [4–5] **	4 [4–5] *	0.14
11. The inhaler can be easily prepared before the first use	4 [4–5]	5 [4–5]	<0.05	5 [4–5] *	5 [4–5] *	0.03
12. The inhaler has additional features confirming correct medication intake (e.g., taste, sound, dose counter)	4 [3–4]	4 [3–5]	<0.05	5 [4–5] **	5 [4–5] *	0.05
13. Patients are easy to train on inhaler use	4 [3–5]	4 [4–5]	<0.05	5 [4–5] **	5 [4–5] *	0.67
14. The medication is properly inhaled regardless of the patient’s ability to take a deep breath	5 [3–4.75]	4 [3–5]	0.18	4 [3–5] *	4 [4–5]	0.86
15. Can be used by patients with limited motor capabilities to operate the inhaler	4 [3–4]	4 [3–5]	<0.05	4 [4–5] **	4 [4–5] **	0.05
16. The inhaler generates the appropriate airflow resistance	4 [3–4.75]	4 [4–5]	<0.05	4 [4–5] **	4 [4–5]	0.96

Please indicate how well each feature related to the use of products for asthma and/or COPD therapy applies to the inhaler you are currently prescribed (CPI)/the inhaler presented (NEXThaler^®^). Use a 5-point scale, where 1 = “Definitely does not apply”, 5 = “Definitely applies”. When providing your answers, please consider features specifically related to the inhaler device itself. * *p* < 0.05, ** *p* < 0.001—for comparing NEXThaler^®^ to CPI within the same group (GPs or specialists).

### 3.4. Patient Assessment of NEXThaler^®^ Versus Currently Used Inhalers

**Figure 4:**
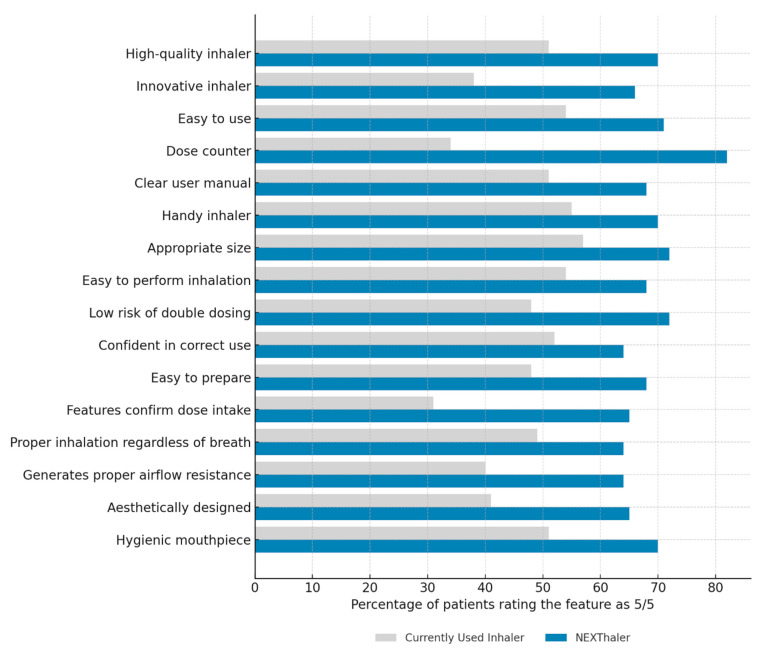
Comparison of patient satisfaction with inhaler characteristics: NEXThaler^®^ vs. currently used inhalers. Percentage of patients who rated each characteristic of their currently used inhaler and NEXThaler^®^ as 5 (highest score) on a 5-point Likert scale.

Patients compared the NEXThaler^®^ with their CUI across 16 predefined characteristics using a five-point Likert scale and by indicating full satisfaction (rating 5/5). As shown in Figure 4, for each feature, a higher proportion of patients gave the maximum score to NEXThaler^®^. This was particularly evident for the dose counter (82% vs. 34%), confirmation features (65% vs. 31%), hygiene of the mouthpiece (70% vs. 51%), and overall quality (70% vs. 51%) (*p* < 0.05).

Statistical analysis confirmed significantly higher median scores for NEXThaler^®^ in 15 out of 16 characteristics (Table 4). These especially included innovation (*p* < 0.0001), presence of a dose counter (*p* < 0.00001), confirmation features (*p* < 0.001), and appropriate airflow resistance (*p* <0.001).

### 3.5. Comparison of NEXThaler^®^ Evaluation Between Patients and Physicians (Table 5, Figure 5)

Both groups evaluated the device highly across nearly all dimensions, with median scores of 5 (on a five-point Likert scale) for the majority of items.

Patients rated the inhaler slightly more favorably than physicians regarding overall satisfaction. A statistically significant difference was observed in how much participants liked the inhaler (*p* < 0.001), with patients providing a more uniformly high rating (median 5 [5–5]) compared to physicians (median 5 [4–5]). Additionally, more patients expressed a willingness to recommend the inhaler (*p* < 0.05) and rated the instructions’ clarity more positively (*p* < 0.05). No significant differences were observed in perceived usability, dose administration, or general preference for daily use. Ratings for technical aspects such as the dose counter, uniqueness, and usability were consistent across groups. However, when asked about the device’s physical size, only 1 out of 96 patients found the inhaler too large, compared to 11 out of 151 physicians (*p* = 0.03), suggesting slightly greater concern among clinicians regarding device portability or ergonomics.

**Table 5:** Comparison of inhaler characteristics: NEXThaler^®^ across patients and doctors.

Inhaler Characteristics	NEXThaler^®^		*p* Values
Median [IQR]	Patients	Doctors	
1. Please rate how much you like this inhaler.	5 [5–5]	5 [4–5]	<0.001
2. Please rate how well this inhaler meets your needs.	5 [4–5]	5 [4–5]	0.06
3. Please rate how unique this inhaler is.	5 [4–5]	4 [4–5]	0.16
4. How likely would you be to use this inhaler daily/in your practice	5 [4–5]	5 [4–5]	0.41
5. How would you rate the usability of this inhaler?	5 [4–5]	5 [4–5]	0.50
6. How would you rate the process of taking a dose with this inhaler?	5 [4–5]	5 [4–5]	0.24
7. How do you rate the dose counter on this inhaler?	5 [4.75–5]	5 [4–5]	0.36
8. Would you recommend the presented inhaler to other patients/doctors, considering its usability	5 [4–5]	4 [4–5]	<0.05
9. How would you rate this inhaler overall?	5 [4–5]	5 [4–5]	0.25
10. Please rate how clear and easy to understand the inhaler’s instructions are.	5 [4–5]	5 [4–5]	<0.05
11. I think the inhaler is too big (N)	1/96	11/151	0.03

Please indicate how well each feature related to the use of products for asthma and/or COPD therapy applies to the inhaler you are currently using/the inhaler presented (NEXThaler^®^). Use a 5-point scale, where 1 = “Definitely does not apply”, 5 = “Definitely applies”. When providing your answers, please consider features specifically related to the inhaler device itself.

**Figure 5:**
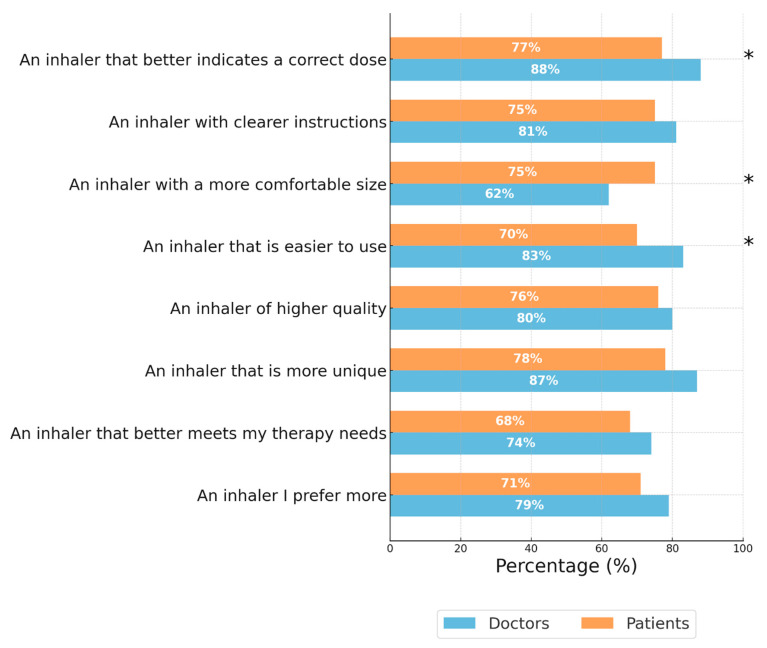
Percentage of doctors and patients who preferred NEXThaler^®^ over their currently prescribed/used inhaler. Blue bars represent the percentage of doctors, while orange bars represent the percentage of patients. Asterisks (*) indicate categories with statistically significant differences (*p* < 0.05) between doctors and patients. Patients and Doctors were asked “Considering both your currently used/prescribed inhaler and the NEXThaler^®^, which one do you prefer based on the following characteristics?”

Figure 5 shows the percentage of doctors and patients who preferred the NEXThaler^®^ over their CPI/CUI across various characteristics. Overall, both doctors and patients showed a high preference for the NEXThaler^®^, with the highest ratings observed for indicating a correct dose (88% doctors, 77% patients), clearer instructions (81% doctors, 75% patients), and ease of use (83% doctors, 70% patients). Statistically significant differences (*p* < 0.05) between doctors and patients were found in three categories: indicating a correct dose, having a more comfortable size, and being easier to use. In these areas, doctors rated the NEXThaler^®^ more favorably in terms of dose indication and ease of use, while patients rated it higher regarding the more comfortable size.

## 4. Discussion

This survey assessed key factors influencing inhaler selection from the perspectives of both patients and physicians, focusing on a direct comparison between NEXThaler^®^ and currently used/prescribed inhalers. The results highlight that ease of inhalation and confidence in correct dose administration are both groups’ primary determinants of inhaler choice. Patients valued device quality, while physicians prioritized reliable delivery regardless of inspiratory effort—highlighting patients’ focus on usability and clinicians’ emphasis on therapeutic precision. Importantly, both groups consistently indicated that factors such as educational materials, cost, and inhaler size were less important in their decision-making.

The present findings are consistent with the Delphi consensus, in which clinicians identified patients’ ability to use the device correctly as the most relevant factor in inhaler selection, underscoring the importance of intuitive devices that require minimal instruction and reduce the burden on healthcare systems [16]. Several surveys have also reported that “ease of performing inhalation” is one of the main determinants influencing patients’ inhaler choice [17,18,19]. However, what patients perceive as “easy to inhale” may vary considerably depending on factors such as device size and shape, clarity of instructions, feedback during inhalation, and the physical effort required to take a dose. Comfort during handling—particularly among individuals with reduced hand strength or arthritis—can also be relevant. In addition, previous experience with inhalers, understanding of treatment, and the quality of training received may strongly influence perceived ease of use [15,19].

In our survey, both patients and physicians ranked ease of inhalation and confidence in administering the correct dose among the top selection criteria, suggesting a shared preference for devices that are practical and straightforward to operate. Patients rated these aspects highly (means 11.09; 95% CI 10.16–12.02 and 9.83; 95% CI 8.87–10.79), similar to physicians (12.35; 95% CI 11.76–12.94 and 15.41; 95% CI 14.78–16.04). Indeed, simple and intuitive operation has been shown to reduce inhalation errors, particularly among older or less educated users, and may contribute to greater satisfaction and adherence [20,21].

In this context, both patients and physicians assigned higher median ratings to NEXThaler^®^ in domains related to ease of use and confidence in correct dose administration (*p* < 0.05), indicating consistent perceptions of usability across groups. These assessments may be linked to features such as the device’s simple three-step operation (open–inhale–close) and feedback confirming correct actuation. Previous comparative studies of DPs, including evaluations of NEXThaler^®^ versus Diskus^®^ and Turbuhaler^®^, have shown that shorter preparation time, straightforward handling, and feedback mechanisms are strongly associated with higher usability and fewer handling errors (*p* < 0.001) [22]. The ease of handling of NEXThaler^®^ was also reported in a study involving 106 older participants (mean age 80 years) with no prior inhaler experience, in which the device required fewer attempts to achieve correct use compared with other inhalers [23].

From a clinical perspective, feedback features such as the dose counter, lactose taste, and the audible “click” of the Breath Actuated Mechanism (BAM) are designed to support correct medication delivery at relatively low inspiratory flow rates (approximately 35 L/min). The audible signal confirms to patients that inhalation has been completed and provides clinicians with an indication of adequate inspiratory effort. Such mechanisms may facilitate correct inhaler technique, while safeguards against double dosing and clear dose tracking support safer device handling [24]. In this survey, these features were associated with higher proportions of respondents assigning the top rating for “confidence in correct use”—from 52% to 64% among patients (*p* = 0.08, indicating a statistical trend), 23% to 49% among GPs (*p* < 0.001), and 46% to 58% among specialists (*p* < 0.05). Nevertheless, about one-third of patients and nearly half of physicians did not express full confidence, indicating that design features alone are insufficient. Emerging digital health solutions, such as smart inhalers that monitor real-time use and provide feedback, may further enhance inhaler technique and user confidence, though their feasibility and effectiveness in clinical practice require further study [25,26].

In our survey, 79% of physicians and 71% of patients indicated a preference for NEXThaler^®^ over their current inhaler, and 68% and 74%, respectively, considered it suitable for meeting therapeutic expectations. These observations are in line with previous findings showing an association between perceived usability and patient preference [22,27]. However, not all comparative studies have confirmed functional differences between inhalers. In a randomized crossover trial comparing NEXThaler^®^ and Turbuhaler^®^, critical error rates were similar, although a larger proportion of participants expressed a preference for NEXThaler^®^ (57.6% vs. 34.5%; *p* = 0.006), suggesting that preference may reflect perceived rather than objective ease of use [28].

While patients in this survey ranked overall device quality among the most important features, physicians placed greater emphasis on reliable drug delivery regardless of inspiratory effort, both ranking these aspects as their second-highest priorities. Reliable drug delivery, although often underestimated by patients, remains clinically relevant, as 22–28% of DPI users make errors due to insufficient inspiratory flow [29]. In line with this, García-Río et al. [30] observed that pulmonologists tend to prioritize high lung deposition and technical performance over patient-related factors such as ease of instruction or awareness of correct inhalation technique. Bridging this gap between technical optimization and patient usability is therefore important. Educational tools—such as placebo inhalers, training replicas with flow indicators, or inspiratory flow meters (e.g., In-Check)—may help improve inhalation technique and reinforce both patient and clinician confidence in correct device use [31,32]

The evaluation of inhalers revealed notable differences between GPs and specialists, reflecting their distinct clinical roles and experience. Specialists, who routinely manage more complex respiratory cases and have greater familiarity with inhalation therapies, tended to assign higher ratings to CPI—particularly for two of the most critical features in inhaler assessment: ease of performing inhalation (median 5 [4–5] vs. 4 [3.25–5], *p* < 0.05) and confidence that patients perform the inhalation correctly (median 4 [4–5] vs. 4 [3–4], *p* < 0.001). In contrast, GPs often face challenges related to time constraints and a broad case mix, which can limit opportunities to focus on inhaler technique and patient instruction. Indeed, the multivariate analysis by Plaza et al. [33] showed that pulmonologists and allergists had about twice the odds (OR 2.33 and 1.98, respectively) of possessing adequate knowledge of inhaled therapy compared with primary care physicians (OR 0.93).

Many GPs rely on basic, familiar inhalers that are not always intuitive, which may increase the risk of misuse. Previous studies have demonstrated substantial gaps in GPs’ skills and knowledge regarding inhaler use. For instance, only 27% were able to correctly demonstrate the use of DPIs, and their understanding was often based on information provided by pharmaceutical representatives rather than structured educational programs [34]. Similarly, in our survey, specialists managed more asthma and COPD patients monthly (150.7 vs. 75.8; *p* < 0.05) and initiated inhaled therapy more frequently (27% vs. 18%; *p* < 0.05), whereas GPs reported higher continuation rates (82% vs. 73%; *p* < 0.05), suggesting that GPs often maintain rather than initiate inhaler therapy, which may reduce their familiarity with device-specific features. Importantly, previous studies have shown that healthcare professionals often overestimate their proficiency in inhaler technique despite evidence to the contrary [35]. A large systematic review found that healthcare providers correctly performed inhalation technique only half as often as patients (15.5% vs. 31%, respectively), highlighting persistent gaps in understanding and inadequate practical knowledge of inhaler use [36].

Despite these challenges, both GPs and specialists provided comparable evaluations of NEXThaler^®^, with no significant differences between groups, indicating broadly consistent perceptions across clinical settings. However, when assessing suitability for patients with physical limitations, both groups assigned relatively low scores to their CPI. Only 13% of GPs awarded the highest Likert rating (median 4 [3–4]), compared with 32% of specialists (median 4 [3–5]). Although ratings for NEXThaler^®^ were somewhat higher (median 4 [4–5], representing a 33% increase among GPs and 51% among specialists), these findings indicate that accommodating the needs of patients with limited motor abilities remains an area requiring further improvement across all inhaler types.

Interestingly, compared with patients, doctors were slightly more cautious in their recommendations (4 [4–5] vs. 5 [4–5], *p* < 0.05) and more often expressed concerns about device size. However, the fact that only 1 patient, compared to 11 doctors, found the inhaler too large (*p* < 0.05) suggests that what clinicians perceive as a limitation may not always align with patients’ experience. This highlights the importance of involving patients in inhaler selection to ensure that choices reflect both clinical criteria and real-world usability.

Given the cross-sectional nature of this survey, the observed relationships between perceived usability, patient preference, and confidence in inhaler use should be interpreted as associations rather than evidence of a causal link. Future research should further explore how patient- and physician-reported preferences translate into clinical outcomes, adherence, and long-term disease control. Larger, prospective studies comparing different inhaler types in real-world settings are needed to validate these findings and assess the clinical relevance of specific design features such as feedback mechanisms or inspiratory flow resistance. By identifying key usability factors and perceptual differences, this survey lays the groundwork for developing more patient-centered devices and educational initiatives for both patients and primary care physicians.

This survey has several limitations that should be acknowledged. Its cross-sectional design captures perceptions at a single point in time, without assessing long-term adherence or clinical outcomes. The evaluation of NEXThaler^®^ was based on visual and instructional demonstrations rather than extended real-world use, which could have influenced the high usability ratings. The survey relied on self-reported data, which carries a risk of recall and response biases. Additionally, the relatively small number of COPD patients limits the depth of subgroup analysis and may underrepresent this group’s specific experiences. Another important limitation is that NEXThaler^®^ was compared to the participants’ currently used or prescribed inhalers as a general category, rather than to a specific reference device, making it difficult to determine how it performs relative to particular inhaler models. The study used the Ipsos Healthcare Panel for participant recruitment, which ensured respondent verification and data quality but may have introduced selection bias. Individuals participating in research panels can differ from the general population of clinicians or patients in motivation or familiarity with digital surveys. Although the demographic distributions in our sample generally aligned with national data for physician specialty and patient age, full representativeness cannot be assumed, which may limit the generalizability of our findings [37]. Furthermore, this study was not blinded, as participants were informed about the NEXThaler^®^ and its features during evaluation. While this approach reflects real-world conditions in which patients and physicians assess inhalers based on usability and design, it may have introduced a positive perception bias. Familiarity with the NEXThaler^®^ brand or the sponsor’s involvement may have influenced participant perceptions despite efforts to minimize such bias. Future studies using blinded or randomized designs could help to better isolate device-related perceptions from brand or presentation. No formal normalization of scores was applied between groups (physicians vs. patients), as the study aimed to capture authentic differences in perception; this choice may, however, limit the comparability of absolute score values between the two groups. Although nonparametric tests were used as the most appropriate approach for ordinal preference data, the absence of multivariable adjustment represents a limitation. Nevertheless, the survey offers important strengths. By incorporating perspectives from both patients and physicians—covering GPs and specialists—it provides a comprehensive view of inhaler preferences and challenges in clinical practice. The MaxDiff analysis provided a systematic evaluation of inhaler feature priorities, and comparisons between NEXThaler^®^ and currently used devices revealed variations in perceived usability and satisfaction.

## 5. Conclusions

Aligning patient and physician perspectives with ongoing developments in inhaler technology remains an important component of optimizing respiratory treatment. Features such as feedback mechanisms and simplified operation—seen in devices like NEXThaler^®^—may enhance user perception and confidence in correct inhaler use, demonstrated by the results of our survey. Nevertheless, since this study reflects single-point, subjective assessments, further research is needed to determine whether these perceptions persist over time and translate into real-world improvements in inhaler technique. The findings also highlight limited familiarity with inhaler characteristics and lower confidence in device selection among GPs, emphasizing the need for ongoing education and practical training to support informed prescribing and effective patient guidance.

## Figures and Tables

**Table 4:** Comparison of inhaler characteristics: currently used inhaler vs. NEXThaler^®^ in patient group.

	CUI	NEXThaler^®^	*p* Values
1. High-quality inhaler	5 [4–5]	5 [4–5]	0.01
2. Innovative inhaler	4 [3–5]	5 [4–5]	<0.0001
3. The inhaler is easy to use	5 [4–5]	5 [4–5]	0.001
4. Dose counter	4 [3–5]	5 [4–5]	<0.00001
5. Includes a clear user manual	4,5 [4–5]	5 [4–5]	<0.05
6. The inhaler is handy	5 [4–5]	5 [4–5]	<0.05
7. The inhaler is the appropriate size	5 [4–5]	5 [4–5]	<0.05
8. Easy to perform inhalation	5 [4–5]	5 [4–5]	<0.05
9. Low risk of taking a double dose of the medication	4 [4–5]	5 [4–5]	<0.01
10. I am confident that I perform inhalation correctly	4,5 [4–5]	5 [4–5]	0.08
11. The inhaler can be easily prepared before the first use	4 [4–5]	5 [4–5]	<0.05
12. The inhaler has additional features confirming correct medication intake (e.g., taste, sound, dose counter)	4 [3–5]	5 [4–5]	<0.001
13. The inhaler delivers medication properly regardless of my ability to take a deep breath	4 [4–5]	5 [4–5]	<0.05
14. The inhaler generates appropriate airflow resistance.	4 [4–5]	5 [4–5]	<0.001
15. It is aesthetically designed.	4 [4–5]	5 [4–5]	<0.01
16. It ensures the possibility of maintaining proper mouthpiece hygiene.	4 [4–5]	5 [4–5]	<0.01

Please indicate how well each feature related to the use of products for asthma and/or COPD therapy applies to the inhaler you are currently using/the inhaler presented (NEXThaler^®^). Use a 5-point scale, where 5 = very, 4 = fairly, 3 = somewhat, 2 = not very, 1 = hardly at all. When providing your answers, please consider features specifically related to the inhaler device itself. CUI—currently used inhaler.

## Data Availability

The data generated and analyzed in the present study are available upon reasonable request from the corresponding author due to concerns regarding privacy and ethical reasons.
